# Supplementary material for: The titin N2A-MARP signalosome constrains muscle longitudinal hypertrophy in response to stretch
Source: bioRxiv. 2025 Jun 25:2025.06.19.660595. Preprint. [Version 1] doi: 10.1101/2025.06.19.660595 (PMC12262232; doi:10.1101/2025.06.19.660595)
Supplement: Supplement 1 [file NIHPP2025.06.19.660595v1-supplement-1.pdf]

bioRxiv preprint doi: https://doi.org/10.1101/2025.06.19.660595; this version posted June 25, 2025. The copyright holder for this preprint (which was not certified by peer review) is the author/funder, who has granted bioRxiv a license to display the preprint in perpetuity. It is made available under aCC-BY 4.0 International license.

Supplemental figures

bioRxiv preprint doi: https://doi.org/10.1101/2025.06.19.660595; this version posted June 25, 2025. The copyright holder for this preprint (which was not certified by peer review) is the author/funder, who has granted bioRxiv a license to display the preprint in perpetuity. It is made available under aCC-BY 4.0 International license.

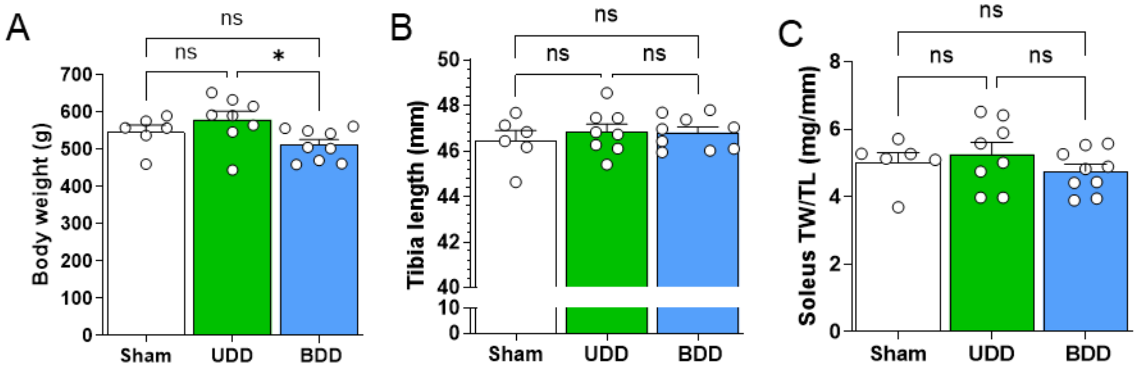

S FIGURE 1. 3-days bilateral diaphragm denervation in rats showed similar body weights compared to sham animals (A; n=6-9/group) and were of similar size based on tibia length (B) and soleus muscle weights (C). Statistical testing by one-way ANOVA and Dunnett's multiple comparisons test.

bioRxiv preprint doi: https://doi.org/10.1101/2025.06.19.660595; this version posted June 25, 2025. The copyright holder for this preprint (which was not certified by peer review) is the author/funder, who has granted bioRxiv a license to display the preprint in perpetuity. It is made available under aCC-BY 4.0 International license.

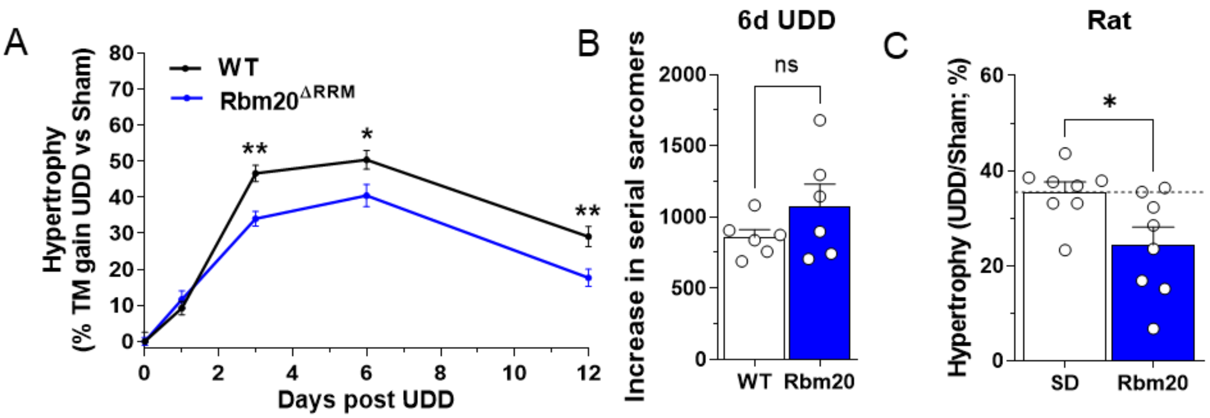

S FIGURE 2. Role of titin stiffness on hypertrophy following UDD. (A) Transient hypertrophy response in Rbm20$^{\Delta RRM}$ mice (more compliant titin) showing a blunted hypertrophy response compared to WT mice, based on percent increase of diaphragm right costal mass relative to sham (n=10-12). (B) Titin-based stiffness does not alter longitudinal hypertrophy response, as both WT and Rbm20$^{\Delta RRM}$ mice show a similar increase in serial sarcomeres following 6-days UDD. Rbm20-KO rat response to 3-days UDD, based on percent increase of diaphragm right costal mass relative to sham (mouse n=10-11, rat n=8) supporting titin-based stiffness regulating muscle hypertrophy similarly across species. Statistical testing by t-test.

bioRxiv preprint doi: https://doi.org/10.1101/2025.06.19.660595; this version posted June 25, 2025. The copyright holder for this preprint (which was not certified by peer review) is the author/funder, who has granted bioRxiv a license to display the preprint in perpetuity. It is made available under aCC-BY 4.0 International license.

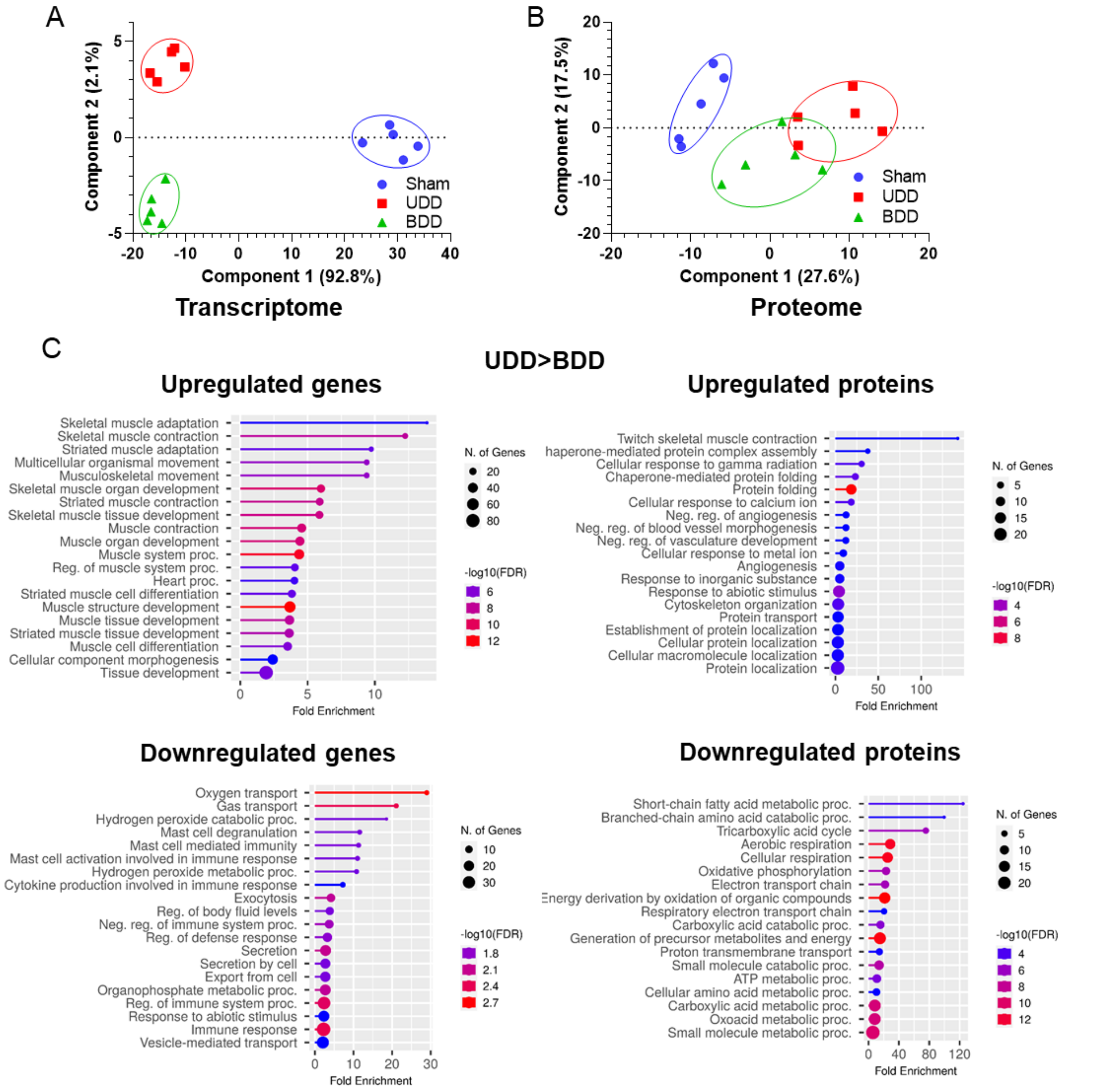

S FIGURE 3. Principal component analysis of the rat 3-day UDD and BDD transcriptome (A) and proteome (B), showing clear separation of groups at the transcript level and overlap of BDD and UDD samples at the protein level. GOterm enrichment of UDD>BDD separated by up- or down-regulated transcriptomes and proteome (C, left and right, respectively) show distinct, yet overlapping cellular processes. Global mass spectrometry was analyzed by ANOVA and corrected for multiple comparisons with false discovery rate with a cut-off at $p<0.05$.

bioRxiv preprint doi: https://doi.org/10.1101/2025.06.19.660595; this version posted June 25, 2025. The copyright holder for this preprint (which was not certified by peer review) is the author/funder, who has granted bioRxiv a license to display the preprint in perpetuity. It is made available under aCC-BY 4.0 International license.

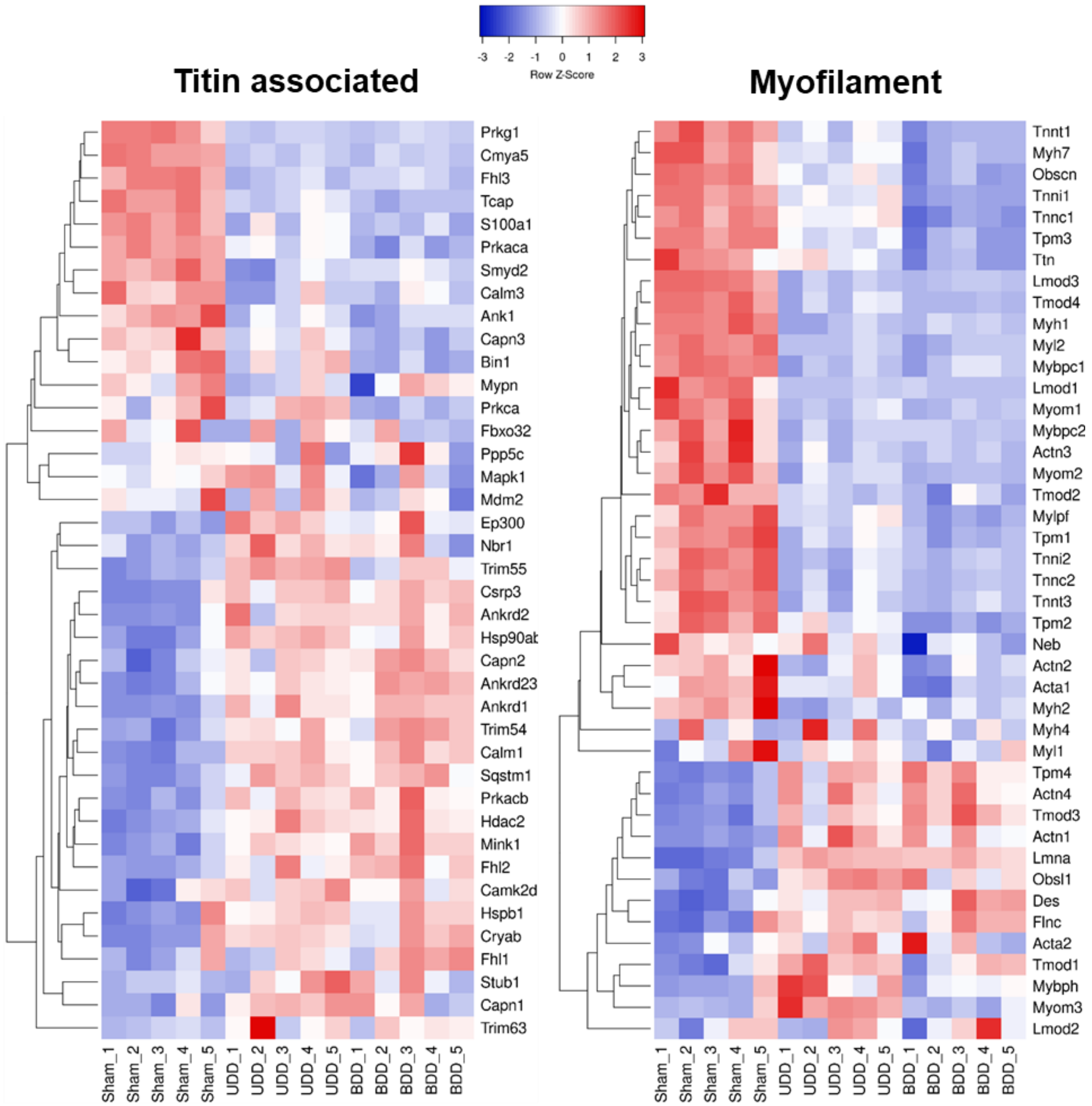

S FIGURE 4. Transcriptome regulation of titin-associated and myofilament genes by RNAseq in rats following 3-days of UDD/BDD. Heatmaps showing similar regulation between UDD and BDD samples (n=4-5; Z-score: red= upregulated, blue= downregulated) at the transcript level for titin-associated and myofilament genes, based on hierarchal clustering.

bioRxiv preprint doi: https://doi.org/10.1101/2025.06.19.660595; this version posted June 25, 2025. The copyright holder for this preprint (which was not certified by peer review) is the author/funder, who has granted bioRxiv a license to display the preprint in perpetuity. It is made available under aCC-BY 4.0 International license.

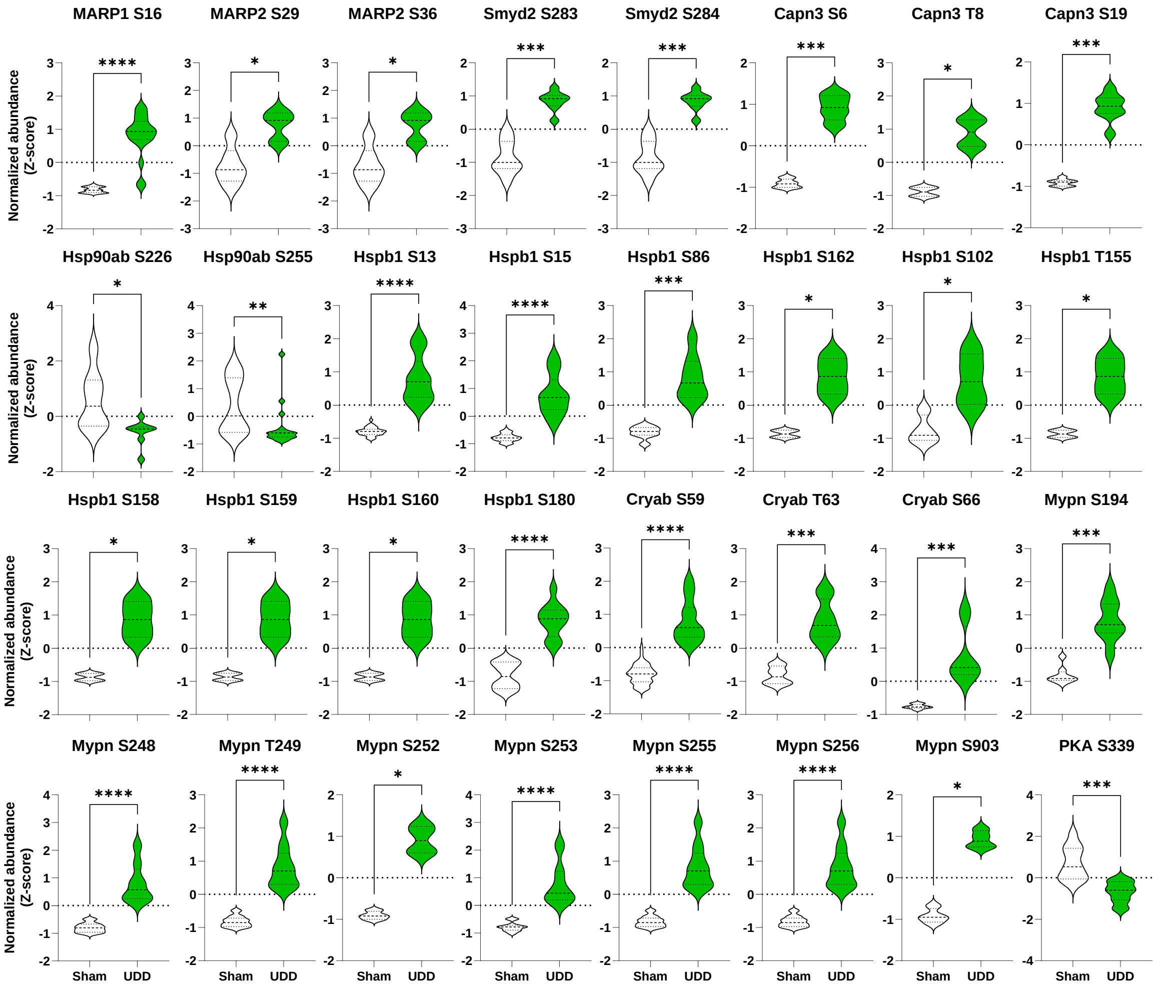

S FIGURE 5. Titin N2A associated protein phosphorylation events at 24-hour UDD. Violin plots of phosphorylation events in N2A-associated proteins following UDD: MARP1 (Transcript: ENSMUST00000237142.2 Ankrd1-205), MARP2 (Transcript: ENSMUST00000026172.3 Ankrd2-201), Smyd2 (Transcript: ENSMUST00000027897.8 Smyd2-201), Capn3 (Transcript: ENSMUST00000028749.15 Capn3-202), Hsp90ab (Transcript: ENSMUST00000024739.14 Hsp90ab1-201), Mypn (Transcript: ENSMUST00000095580.3 Mypn-201), Hspb1 (Transcript: ENSMUST00000005077.7 Hspb1-201), Cryab (Transcript: ENSMUST00000217475.2 Cryab-206) and Prkca/PKA (Transcript: ENSMUST00000005606.8 Prkaca-201). Data represented as Log2 of the normalized abundance with significance determined by Kolmogorov-Smirnov test.

bioRxiv preprint doi: https://doi.org/10.1101/2025.06.19.660595; this version posted June 25, 2025. The copyright holder for this preprint (which was not certified by peer review) is the author/funder, who has granted bioRxiv a license to display the preprint in perpetuity. It is made available under aCC-BY 4.0 International license.

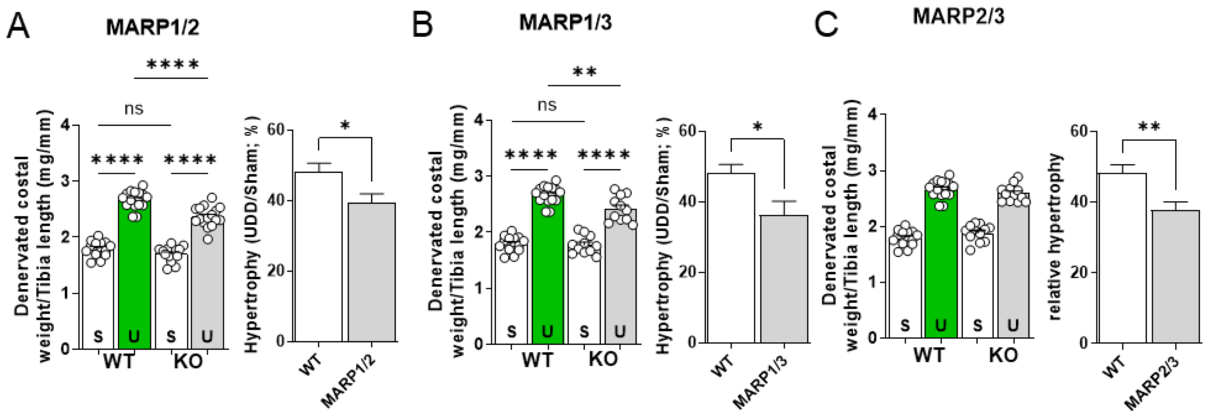

S FIGURE 6. 6-day UDD on double KO mice of MARPs. Double KO of MARP1/2 (A), MARP1/3 (B) and MARP2/3 (C) all showed a reduction in hypertrophy following UDD, suggesting redundancy between the MARPs. Left panel, diaphragm right costal mass normalized to tibial length and right panel, percentual increase in right costal mass relative to sham. S= Sham, U= UDD (n=10-12).
